# Treatment with a copper-selective chelator causes substantive improvement in cardiac function of diabetic rats with left-ventricular impairment

**DOI:** 10.1186/1475-2840-12-28

**Published:** 2013-01-31

**Authors:** Jun Lu, Beau Pontré, Stephen Pickup, Soon Y Choong, Mingming Li, Hong Xu, Gregory D Gamble, Anthony RJ Phillips, Brett R Cowan, Alistair A Young, Garth JS Cooper

**Affiliations:** 1 School of Biological Sciences, University of Auckland, Auckland, New Zealand; 2 Maurice Wilkins Centre for Molecular Biodiscovery, University of Auckland, Auckland, New Zealand; 3 Centre for Advanced MRI, University of Auckland, Auckland, New Zealand; 4 Department of Physiology, University of Auckland, Auckland, New Zealand; 5 Department of Medicine, University of Auckland, Auckland, New Zealand; 6 Department of Surgery, University of Auckland, Auckland, New Zealand; 7 Small Animal Imaging Facility, Department of Radiology, University of Pennsylvania School of Medicine, Philadelphia, PA, USA; 8 Department of Interdisciplinary Studies, Faculty of Health and Environmental Sciences, Auckland University of Technology, Auckland, New Zealand; 9 College of Chemistry and Chemical Engineering, Shenzhen University, Shenzhen, Guangdong Province, Peoples’ Republic of China; 10 Centre for Advanced Discovery and Experimental Therapeutics, Manchester Biomedical Research Centre, University of Manchester, Manchester, M13 9PT, United Kingdom; 11 School of Biomedicine, Faculty of Medical and Human Sciences, University of Manchester, Manchester, United Kingdom; 12 Department of Pharmacology, Medical Sciences Division, University of Oxford, Oxford, United Kingdom

**Keywords:** Heart failure, Cardiovascular disease, Cardiac output, Complications of diabetes, Diabetic cardiomyopathy, Diastolic function, Cardiac magnetic resonance imaging, Left-ventricular ejection fraction, Left-ventricular end systolic volume, Left-ventricular end diastolic volume, Selective copper chelation, Systolic function, Experimental therapeutics

## Abstract

**Background:**

Defective copper regulation is implicated as a causative mechanism of organ damage in diabetes. Treatment with trientine, a divalent-copper-selective chelator, improves arterial and renal structure/function in diabetes, wherein it also ameliorates left-ventricular (LV) hypertrophy. However, direct in vivo evidence that trientine can improve cardiac function in heart failure has hitherto been lacking.

**Methods:**

To determine whether trientine treatment could improve in vivo outcome, we measured cardiac function in groups of trientine-treated diabetic (TETA-DIA), non-drug-treated diabetic (DIA) and sham-treated control (SHAM) rats, by using in vivo high-field cardiac magnetic-resonance imaging (cMRI) and an ex vivo isolated-perfused working heart method. Forty age-matched animals underwent a cMRI scan after which 12 were randomized to the SHAM group and 28 underwent streptozotocin-injection; of these, 25 developed stable diabetes, and 12 were then randomized to receive no treatment for 16 weeks (DIA) and the other 13 to undergo 8-weeks’ untreated diabetes followed by 8-weeks’ drug treatment (TETA-DIA). Animals were studied again by cMRI at 8 and 16 weeks following disease induction, and finally by measurement of ex vivo cardiac function.

**Results:**

After eight weeks diabetes, rats (DIA/TETA-DIA) had developed significant impairment of LV function, as judged by impairment of ejection fraction (LVEF), cardiac output (CO), and LV mass (LVM)/body-mass (all *P <* 0.001), as well as other functional indexes. LVEF, CO (both *P <* 0.001) and the other indexes deteriorated further at 16 weeks in DIA, whereas trientine (TETA-DIA) improved cardiac function by elevating LVEF and CO (both *P <* 0.001), and also partially reversed the increase in LVM/body-mass (*P <* 0.05). In ex vivo hearts from DIA, the CO response to increasing preload pressure was deficient compared with SHAM (*P <* 0.001) whereas the preload-CO relationship was significantly improved in TETA-DIA animals (*P <* 0.001).

**Conclusions:**

Trientine treatment significantly improved cardiac function in diabetic rats with substantive LV impairment. These results implicate impaired copper regulation in the pathogenesis of impaired cardiac function caused by diabetic cardiomyopathy, and support ongoing studies of trientine treatment in patients with heart failure.

## Introduction

Cardiovascular disease is the leading cause of morbidity and mortality in diabetes, but effective treatments for established heart failure in diabetes are limited [1-3]. The prognosis of heart failure is particularly poor in patients with type-2 diabetes (T2DM)^a^, the most common form of this disease [4]. This poor prognosis persists in spite of best available treatment with existing classes of medications including glucose-lowering, antihypertensive, and lipid-lowering drugs, as well as beta-blockers, angiotensin-converting enzyme inhibitors and angiotensin II receptor blockers (ARBs) amongst others. Therefore, new and improved therapeutic approaches for heart failure in diabetes are urgently required.

We have previously identified a pathogenetic disorder of copper regulation that occurs in both type-1 diabetes (T1DM) and T2DM [5,6], and have shown that it is a probable cause of the cardiovascular and renal complications of diabetes [5,7-9] and a new target for pharmacological intervention [5,6,10]. We have also shown that trientine (triethylenetetramine dihydrochloride) [10], acts in vivo as a Cu(II)-selective chelator that can prevent or ameliorate cardiovascular and renal disease in diabetic rats [5,8,11]. Trientine exerts its effects in diabetic animals and patients without lowering blood pressure or blood glucose [5,12].

We have extended our studies to patients with T2DM [6,10,12,13], in whom trientine treatment significantly improves LV hypertrophy [12] and indexes of metabolic regulation [6].

Triethylenetetramine (also named TETA or trien) is the pharmacologically active moiety in trientine. Its structure closely resembles those of the endogenous polyamines, spermine and spermidine [14,15]. Trientine is a well-tolerated orally-active drug which acts as a Cu(II)-selective chelator in humans [6,10] and has been employed for more than two decades for the second-line treatment of Wilson’s disease in penicillamine-intolerant patients [16-18]. It has been linked to occasional cases of anemia [19] and thrombocytopenia [20] in patients with Wilson’s disease.

Diabetic animals show signs consistent with dysregulation of systemic copper homeostasis. These manifest in organs including the heart, arteries, kidneys and nerves [21]. In normal physiology, copper is present in one of two valence states: univalent copper, Cu(I), which is said to comprise ~95% of total body copper and is the predominant intracellular form, and Cu(II) which comprises the remainder and is mainly present in the extracellular space; in quoting this relative distribution of copper valence states, we follow Fraústo da Silva and Williams [22]. Infusion of trientine has revealed that systemic Cu(II) is significantly elevated in diabetic rats [5,8,9]. Nonclinical [5] and clinical studies [6,12,14] have shown that diabetic rats and patients display signs of a systemic copper overload state, which is characterized by the following characteristics: elevations in basal urinary copper excretion; elevated copper balance; increased urinary Cu(II) excretion following administration of trientine; and increased circulating superoxide dismutase activity. Furthermore, levels of Cu(II) extracted from the coronary arteries by trientine infusion are also significantly increased in diabetic rats [5]. Trientine binds selectively to Cu(II) but not to Cu(I) so elevations in trientine-chelatable copper are consistent with elevated Cu(II) [5,21].

In vivo data demonstrating trientine-mediated improvements in cardiac function in diabetic animals with heart failure have hitherto been lacking. Here we report results of a study in which we have tested the hypothesis that orally-administered trientine can improve in vivo cardiac function in diabetic rats with established heart failure.

## Materials and methods

### Animals and treatments

This study was approved by the University of Auckland Animal Ethics Committee. Its performance was consistent with principles described in the Guide for the Care and Use of Laboratory Animals [23] and the ARRIVE guidelines for the reporting of animal research [24].

**Figure 1:**
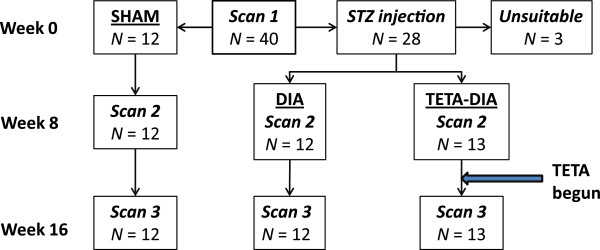
Flow chart illustrating the make-up and disposition of the study groups during the 16-week course of the in vivo part of this study.

Forty male Wistar rats, whose age was between six and seven weeks and whose average body-weight was 256 ± 37 g mean (± SD) at commencement, were sourced from the Integrated Physiology Unit (School of Biological Sciences, University of Auckland) and entered into the study. They were housed (12:12-h light–dark cycle; temperature 22.5°C [range: 20–26]; humidity 50-70%) in like-pairs with ad libitum access to water and food (Teklad 2018, Harlan Laboratories, Placentia, CA). Study-group characteristics were as summarized in Table 1, and the work-flow followed during the in vivo part of the study was as shown in Figure 1.

**Table 1:** Relevant characteristics and between-group comparisons at baseline (Week 0)

**Variable**	**Control (SHAM)**	**Diabetic (DIA)**	**TETA-treated Diabetic (TETA-DIA)**	***P-*value **^**†**^
**Strain**	Wistar	Wistar	Wistar	-
**Age (weeks)**	6-7	6-7	6-7	-
Number (% male)*	12 (100)	12 (100)	13 (100)	-
**Body-weight (g)**	271 (255–287)	280 (257–302)	255 (239–271)	NS
**Blood glucose (mM)**	5.1 (4.9-5.2)	6.0 (5.7-6.3) ^a^	6.7 (5.9-7.5) ^a^	< 0.001
**Heart rate (bpm)**	380 (370–389)	376 (364–388)	393 (381–401)	0.059
**LVM (mg)**	569 (525–614)	566 (521–610)	528 (493–562)	NS
**LVM/BW (mg/g)**	2.12 (1.89-2.35)	2.04 (1.87-2.22)	2.08 (1.95-2.21)	NS
**CO (ml/min)**	133 (120–146)	139 (125–152)	137 (130–140)	NS
**CO/BW (ml/min.g)**	0.50 (0.44-0.55)	0.50 (0.43-0.58)	0.54 (0.50-0.59)	NS
**CO/LVM (ml/min.mg)**	0.24 (0.21-0.26)	0.25(0.23-0.26)	0.26(0.24-0.29)	NS
**LVEF (%)**	72.2 (69.6-74.9)	74.7 (72.9-76.5)	73.4 (71.4-75.5)	NS
**LVEDV (μl)**	485 (446–525)	494 (440–549)	476 (456–496)	NS
**LVEDV/LVM (μl/mg)**	0.86 (0.78-0.94)	0.87 (0.82-0.93)	0.91 (0.85-0.98)	NS
**LVESV (μl)**	134 (120–149)	125 (108–143)	126 (116–136)	NS
**LVESV/LVM (μl/mg)**	0.28 (0.25-0.30)	0.25 (0.24-0.27)	0.27 (0.25-0.29)	NS
**Stroke Vol (μl)**	352 (317–386)	369 (329–410)	350 (330–370)	NS
**Stroke Vol/LVM (μl/mg)**	0.62 (0.56-0.69)	0.65 (0.61-0.70)	0.67 (0.61-0.73)	NS

Variables with bracketed numbers are means (± 95% CI) except for number (%) *: ^a^, significantly greater than corresponding SHAM value. ^†^One-way ANOVA with post-hoc Tukey’s tests as indicated. Abbreviation: bpm, beats/min; NS, not significant; -, not tested.

After the first cMRI scan (at Week 0), 28 animals were randomly selected to receive a single intravenous tail-vein injection of streptozotocin (STZ, Sigma; 55 mg/kg body-weight) in isotonic saline as described previously [5], and tail-vein blood-glucose values monitored weekly thereafter. The remaining 12 rats were designated as healthy controls (SHAM) and sham-treated by injection of an equivalent volume of isotonic saline (vehicle) only. Twenty-five of the 28 STZ-treated rats became stably diabetic, as determined by repeated blood glucose values > 11 mM. Of these, 12 were then randomized to the untreated diabetic group (DIA) and 13 to the TETA-treated diabetic group (TETA-DIA). Data from the 37 animals in the 3 study groups, comprising 12 controls (SHAM), 12 untreated-diabetic (DIA) and 13 drug-treated diabetic (TETA-DIA) were collected and have been analysed herein.

Eight weeks after diabetes induction, the 37 rats underwent a second cMRI study. Thereafter, the TETA-DIA animals were continuously treated with trientine (Fluka) at 20 mg/day-rat via their drinking water for the subsequent 8-week period, whereas the DIA rats receive water without drug. At the end of Week 16, all rats underwent a third cMRI scan after which they were anesthetized (isoflurane), heparinized (200 I.U./kg i.v.), and their hearts excised. Ex vivo indexes of cardiac function were then determined in isolated-perfused working heart preparations by using established methods [5,25].

### In vivo cardiac magnetic resonance imaging

Cardiac MR images were acquired just before the time of diabetes induction during experimental Week 0, and at the ends of Weeks 8 and 16 by using a Varian 4.7-Tesla horizontal-bore magnet controlled by a Unity Inova spectrometer (Palo Alto, CA, USA). Animals were anesthetized with 2-4% (v/v) isoflurane in air adjusted according to respiratory monitoring, with subsequent placement of electrocardiogram (ECG)-, respiration- and temperature-monitoring electrodes (SAII, Stony Brook, NY), and mounting in a 72-mm inner-diameter circularly-polarized bird-cage coil (M2M Imaging, Cleveland, OH). Core body temperature was maintained at 35-38°C by directing a regulated stream of warm air over the animal, and heart rate was recorded throughout the study.

Following acquisition of scout images, a cardiac- and respiration-gated T_1_-weighted gradient-echo cine study was acquired in the cardiac short-axis orientation spanning from the apex to the base of the LV (Repetition time, TR = 2 × R-R interval, ~ 280–360 msec; echo time, TE = 2.2 msec, cardiac phases = 20, flip angle = 20°, slices = 6, thickness = 2 mm, averages = 2, field of view = 60 × 60 mm, matrix = 128 × 128, gap between slices = 0.6-1.0 mm according to the size of the heart). A second cine study was then performed in the cardiac long-axis view using the same acquisition parameters (slices = 3, and oriented to give 2-, 3- and 4-chamber views). Cardiac short-axis images were then generated by using the multiecho Dixon fat and water separation method [26,27] (TR = 1000 msec, TE = 20 msec and matrix = 256 × 128).

Left-ventricular end-diastolic volume (LVEDV), left-ventricular end-systolic volume (LVESV) and left-ventricular mass (LVM) were determined by manual contouring of the endocardial and epicardial borders of the short axis cine images using ImageJ (National Institutes of Health, v1.44i; http://rsb.info.nih.gov/ij), and LV-wall volume and LVM were calculated by slice summation (LVM = (epicardial – endocardial volume) × density of 1.05 g/ml) as previously described [5,28]. A similar method was used to estimate the cardiac-fat volume in the Dixon images by using ImageJ. Left-ventricular ejection fraction (LVEF) was calculated as (LVEDV-LVESV)/LVEDV and LV cardiac output (CO) as (LVEDV-LVESV) × heart rate.

### Isolated-perfused working-heart model

Ex vivo measurements of systolic function were performed following excision of isolated-perfused working hearts at the end of Week 16 in the three treatment groups, by using our previously described methods [5,7]. In brief, excised hearts were immersed in Krebs-Henseleit bicarbonate buffer (KHB) at 4°C. Retrograde (Langendorff) perfusion was initially established (KHB, 37°C, gassed with O_2_:CO_2_ 95:5 vol/vol), after which working-mode perfusion was instituted at physiological values of preload pressure and heart-rate (preload, 10 cmH_2_O; afterload, 55.9 mmHg; with pacing at 300 beats/min (Digitimer)) [5,29]. Intra-chamber LV pressure (SP855; AD Instruments), aortic pressure (PX23XL, Stratham Gould), and aortic (Transonic T206) and coronary flows were measured (SP855) (Powerlab16s, ADI) and CO calculated. Atrial filling pressure was then decreased, to 5 cmH_2_O, and thereafter increased, in seven steps of 2.5 cmH_2_O to 20 cmH_2_O [final], and 1-min averages were extracted.

### Statistical analysis

Data in tables have generally been expressed as means (± 95% CI). The significance of between-group differences at specific time-points has been assessed by the fitting of ANOVA models (with post-hoc Tukey’s tests as required) and of time-dependent effects by linear mixed-effects modelling (LME) (Splus v8.1; Spotfire, TIBCO, Somerville, MA). *P-*values of < 0.05 have been adjudged significant. *P-*values of 0.05 ≤ *P* ≤ 0.10 have been included in tables if they occurred.

## Results

### Effects of diabetes and trientine treatment on blood glucose and body-weight

Work-flows were as shown in Figure 1 and data and statistical analysis as summarized in Tables 1, 2, 3, 4. Descriptive data were comparable in all groups at baseline, except for a modest albeit statistically-significant difference in blood glucose levels (Table 1).

**Table 2:** Between-group comparisons at Week 8: effects of diabetes on cardiac function directly before commencement of TETA treatment

**Variable**	**Control (SHAM)**	**Diabetic (DIA)**	**TETA-treated diabetic** **(TETA-DIA)**	***P-*value **^**†**^
**Body-weight (g)**	440 (425–455)	319 (298–340) ^a^	326 (305–347) ^a^	< 0.0001
**Blood glucose (mM)**	5.1 (4.7-5.6)	29.5 (27.0-32.1) ^a^	28.1 (25.3-30.8) ^a^	< 0.0001
**Heart Rate (bpm)**	401 (391–411)	322 (311–332) ^a^	343 (335–352) ^a, b^	< 0.0001
**LVM (mg)**	776 (731–820)	687 (651–723) ^a^	710 (668–751) ^a^	0.006
**LVM/BW (mg/g)**	1.76 (1.68-1.85)	2.17 (2.01-2.33) ^a^	2.19 (2.07-2.31) ^a^	< 0.0001
**CO (ml/min)**	200 (194–206)	135 (127–144) ^a^	135 (129–140) ^a^	< 0.0001
**CO/LVM (ml/min.mg)**	0.26 (0.24-0.28)	0.20 (0.18-0.21) ^a^	0.19 (0.18-0.20) ^a^	< 0.0001
**LVEF (%)**	73.7 (71.0-76.4)	68.2 (67.0-69.5) ^a^	66.3 (64.5-68.0) ^a^	< 0.0001
**LVEDV (μl)**	679 (651–706)	617 (584–651) ^a^	591 (572–610) ^a^	< 0.0001
**LVEDV/LVM (μl/mg)**	0.88 (0.84-0.92)	0.90 (0.85-0.95)	0.84 (0.79-0.89)	NS
**LVESV (μl)**	179 (157–202)	196 (183–209)	199 (189–210)	NS
**LVESV/LVM (μl/mg)**	0.26 (0.24-0.29)	0.32 (0.31-0.33) ^a^	0.34 (0.32-0.35) ^a^	< 0.0001
**Stroke Vol (μl)**	500 (480–520)	421 (396–446) ^a^	392 (374–411) ^a^	< 0.0001
**Stroke Vol/LVM (μl/mg)**	0.65 (0.61-0.68)	0.61 (0.58-0.65)	0.56 (0.52-0.59) ^a, b^	0.001

Values are means (± 95% CI). ^†^ One-way ANOVA with post-hoc Tukey’s tests as necessary: ^a^, significantly different from corresponding SHAM value; ^b^, significantly different from corresponding DIA value. Abbreviations: bpm, beats per minute; NS, not significant.

**Table 3:** Between-group comparisons at Week 16: effects of 8-weeks TETA treatment on cardiac function in diabetic cardiomyopathy

**Variable**	**Control (SHAM)**	**Diabetic (DIA)**	**TETA-treated diabetic (TETA-DIA)**	***P-*value **^**†**^
**Body-weight (g)**	489 (466–511)	313 (283–344) ^a^	346 (323–368) ^a^	< 0.0001
**Blood glucose (mM)**	4.7 (4.4-5.1)	28.8 (27.2-30.4) ^a^	28.0 (25.7-30.4) ^a^	< 0.0001
**Heart Rate (bpm)**	396 (384–408)	317 (306–328) ^a^	336 (326–346) ^a, b^	< 0.0001
**LVM (mg)**	785 (744–826)	732 (698–767)	737 (688–767)	0.049
**LVM/BW (mg/g)**	1.61(1.51-1.71)	2.38 (2.18-2.57) ^a^	2.12 (1.98-2.27) ^a, b^	< 0.0001
**CO (ml/min)**	222 (213–232)	130 (123–136) ^a^	159 (151–167) ^a, b^	< 0.0001
**CO/LVM (ml/min.mg)**	0.28 (0.26-0.30)	0.18 (0.17-0.19) ^a^	0.22 (0.21-0.23) ^a, b^	< 0.0001
**LVEF (%)**	72.9 (70.7-75.1)	62.6 (60.6-64.6) ^a^	71.1 (68.8-73.4) ^b^	< 0.0001
**LVEDV (μl)**	770 (753–788)	656 (628–683) ^a^	666 (644–687) ^a^	< 0.0001
**LVEDV/LVM (μl/mg)**	0.99 (0.94-1.03)	0.90 (0.85-0.95) ^a^	0.92 (0.87-0.97)	0.030
**LVESV (μl)**	209 (192–225)	246 (229–262) ^a^	193 (177–209) ^b^	< 0.0001
**LVESV/LVM (μl/mg)**	0.27 (0.25-0.29)	0.37 (0.35-0.39) ^a^	0.29 (0.27-0.31) ^a, b^	< 0.0001
**Stroke Vol (μl)**	562 (540–585)	410 (388–433) ^a^	473 (452–495) ^b^	< 0.0001
**Stroke Vol/LVM (μl/mg)**	0.72 (0.68-0.76)	0.56 (0.53-0.59) ^a^	0.65 (0.62-0.69) ^a, b^	< 0.0001

Values are means (± 95% CI). ^†^ One-way ANOVA with post-hoc Tukey’s tests as necessary: ^a^, significantly different from corresponding SHAM value; ^b^, significantly different from corresponding DIA value. Abbreviation: bpm, beats per minute.

**Table 4:** Summary of analysis by linear mixed-effects modelling of the time-dependent effects of trientine treatment between Weeks 8 and 16 on indexes of cardiac function in diabetic cardiomyopathy

**Variable**	**Treatment**	**Week**	**Treatment:Week**
**Body-weight (g)**	NS	NS	0.035
**Blood glucose (mM)**	NS	NS	NS
**Heart rate (bpm)**	0.098	NS	NS
**LVM (g)**	0.023	NS	0.046
**LVM/BW**	0.096	0.0057	0.0077
**CO (ml/min)**	0.0009	NS	< 0.0001
**CO/LVM (ml/min.mg)**	0.0001	0.0003	< 0.0001
**LVEF (%)**	< 0.0001	< 0.0001	< 0.0001
**LVEDV (μl)**	0.018	0.0003	0.0091
**LVEDV/LVM (μl/mg)**	0.0041	NS	0.0013
**LVESV (μl)**	0.0013	< 0.0001	< 0.0001
**LVESV/LVM (μl/mg)**	< 0.0001	< 0.0001	< 0.0001
**Stroke Vol (μl)**	< 0.0001	0.10	< 0.0001
**Stroke Vol/LVM (μl/mg)**	< 0.0001	0.0002	< 0.0001

Numbers are *P-*values derived from the fitting of LME models of the general form (Variable ~ [treatment + week + treatment:week]) for each of the indicated variables, wherein individual rats were considered to generate random effects. Abbreviations: bpm, beats per minute; NS, not significant; Treatment:Week, treatment-week interaction term.

**Figure 2:**
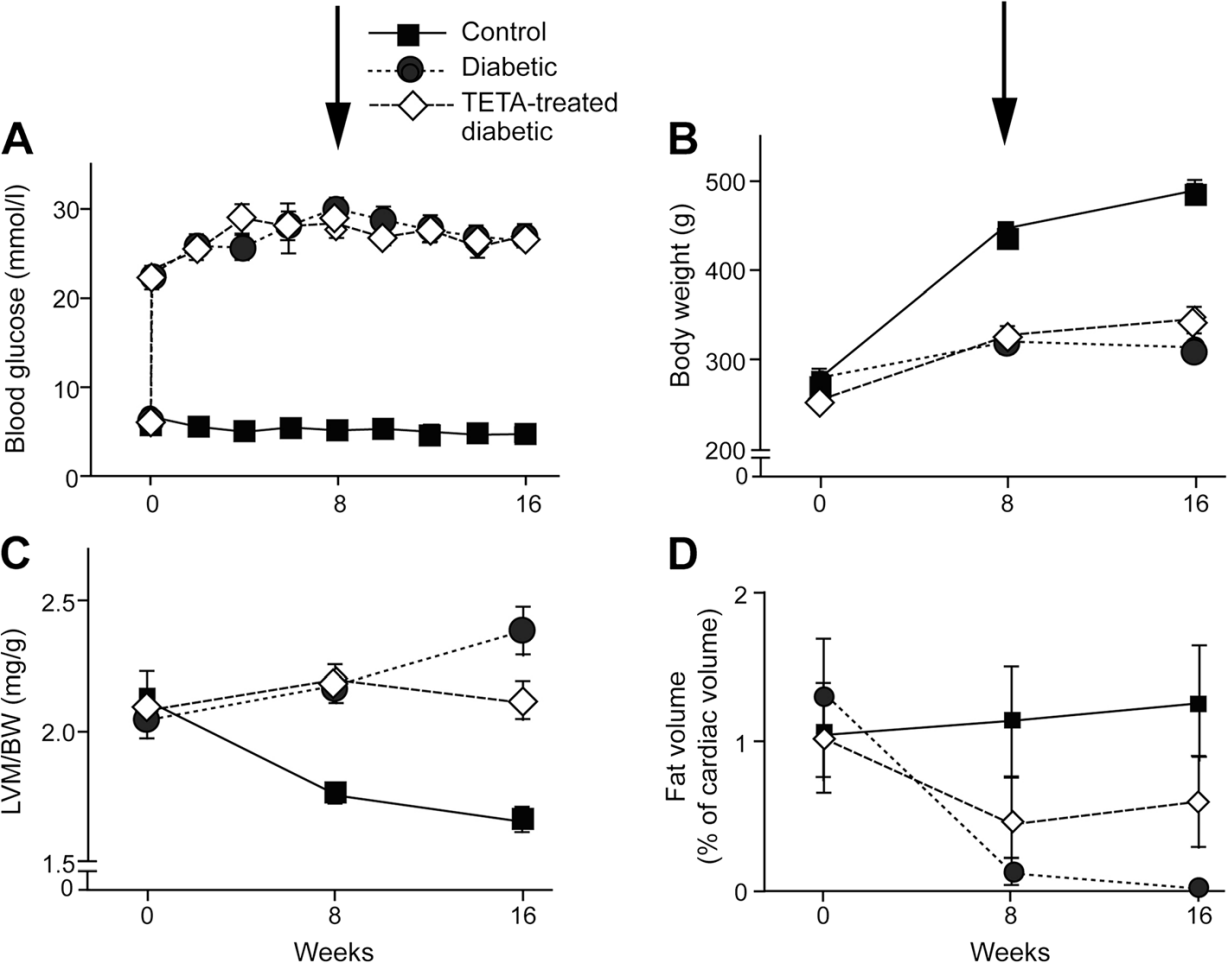
**Time- and treatment-dependent changes in blood glucose concentrations, body-weight, and indexes of LV size and composition during the 16-week experimental period in groups of control (n = 12), untreated-diabetic (n = 12) and trientine-treated diabetic (n = 13) adult male rats.** Drug treatment was initiated after the measurements of Week 8 (arrowed in **A**, **B**). **A**, Blood glucose concentrations; **B**, Body-weight; **C**, Ratio of LV mass to body-weight (LVM/BW); **D**, Percentage of cardiac fat volume to myocardial volume in the LV. Abbreviation: TETA, triethylenetetramine.

All diabetic rats that were randomized remained diabetic throughout the 16-week experimental period (Figure 2A, Tables 2, 3, 4). At Weeks 8 and 16, DIA and TETA-DIA rats had much higher glucose values than SHAM (*P <* 0.0001) and trientine had no effect on glucose levels (Tables 3 and 4).

Body-weight did not differ between experimental groups at baseline (Table 1). Sham-treated control rats gained significant body-weight during the 16-week experimental period whereas weight-gain in diabetic rats was much more limited (Figure 2B and Tables 2, 3, 4). SHAM rats had significantly higher body-weights than DIA and TETA-DIA animals at Weeks 8 and 16 (both *P <* 0.001; Tables 2, 3, 4). Trientine had a modest effect on the body-weight:time interaction in diabetic animals consistent with the slight amelioration of diabetes-evoked impairment of weight gain (*P =* 0.035, LME; Table 4).

### Diabetes-evoked left-ventricular impairment

Diabetic rats developed LV impairment that was evident at Week 8. This was characterised by impairment in indexes of cardiac structure/function, including LVM/body-weight, CO, CO/LVM, LVEF, LVEDV, LVESV/LVM, stroke volume and stroke volume/LVM (all *P <* 0.0001 except the last, which was *P =* 0.001) (Table 2). The effects of diabetes were comparable in DIA and TETA-DIA groups, save that the lowering of heart rate was more severe in DIA, whereas the depression of stroke volume was more pronounced in TETA-DIA (Table 2). LV function in DIA deteriorated further at Week 16 (Tables 2, 3, 4).The between-group difference in diabetes-induced bradycardia persisted at Week 16 and trientine had no effect on heart rate (Tables 2, 3, 4).

### Effects of diabetes and trientine on left-ventricular mass and the percentage of cardiac fat volume to myocardial volume

**Figure 3:**
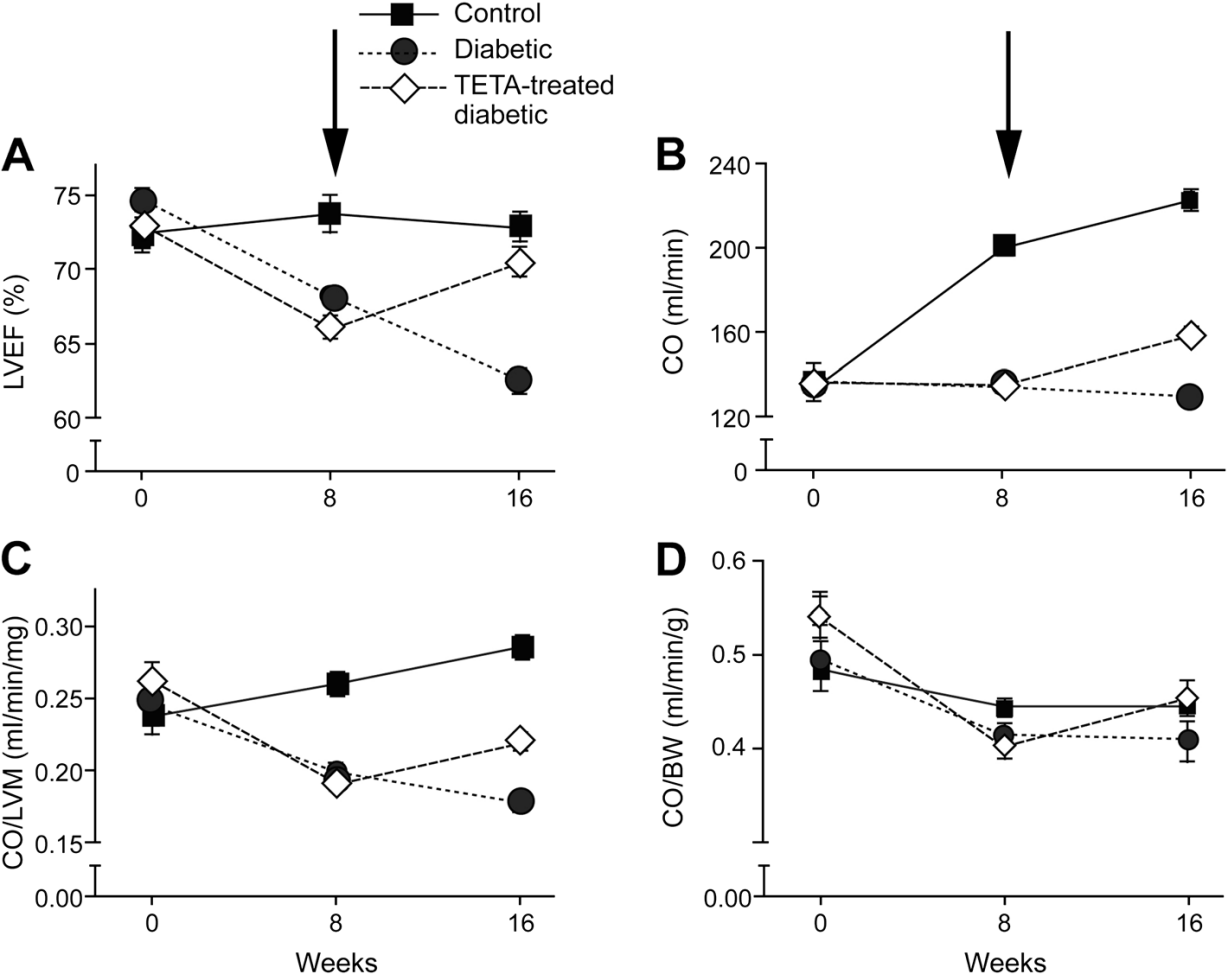
**Time- and treatment-dependent changes in cMRI-derived cardiac functional parameters during the 16-week experimental period in groups of control (n = 12), untreated-diabetic (n = 12) and trientine-treated diabetic (n = 13) adult male rats.** Drug treatment was initiated after the measurements of Week 8 (arrowed in **A**, **B**): **A**, LV ejection fraction (LVEF); **B**, LV cardiac output (CO); **C**, Ratio of CO/LV mass (CO/LVM); **D**, ratio of CO to body-weight (CO/BW). Abbreviation: TETA, triethylenetetramine.

We have employed LVM normalized to BW (LVM/BW) as an index of body-mass, as in previous studies [5]. Some workers prefer the indexing of LVM to tibial length, but others prefer LVM/BW. The improved discrimination of the effects of drug treatment on LVM/BW as opposed to unindexed LVM observed in this study (Tables 2, 3, 4) provides support for the use of LVM/BW. Here, variables pertaining to cardiac function, including CO, LVEDV, LVESV and stroke volume, have been normalized to LVM, and our evidence clearly indicates that CO/LVM is preferable to CO/BW as a normalization procedure (Tables 1, 2, 3, 4, Figure 3).

Changes in the LVM-to-body-weight ratio (LVM/BW) [30] over the experimental period are shown (Figure 2C and Tables 1, 2, 3, 4). At the outset, all three experimental groups had similar LVM/BW ratios. As they aged, the LVM/BW ratio in SHAM rats fell progressively. By contrast, the LVM/BW ratio in both diabetic groups had increased at Week 8. At Week 16, the LVM/BW values in DIA rats had increased further, consistent with worsening of the relative LV hypertrophy (*P <* 0.001). Although TETA-DIA rats had significantly lower LVM/BW values than DIA at Week 16 (*P <* 0.05), their values were still significantly higher than those of SHAM animals (*P <* 0.001). Furthermore, the LVM/BW:time interaction term for trientine treatment was significant (LME, *P =* 0.0077). Trientine thus ameliorated the relative cardiac hypertrophy in diabetic rats.

Diabetes significantly lowered the percentage of cardiac fat volume to myocardial volume in the LV (*P <* 0.001, Figure 2D), but trientine treatment did not alter this variable. LV fat content in these diabetic rats was significantly lower than that in the non-diabetic controls, consistent with the severely insulin-deficient model of diabetes employed in this study.

### Effects of trientine treatment on left-ventricular function

Eight weeks diabetes significantly lowered many indexes of LV function (Table 2) including LVEF values (*P <* 0.0001, Figure 3A), which worsened still further after the second eight-week period of untreated disease (*P <* 0.0001). By contrast, eight weeks oral trientine treatment significantly improved LVEF (*P <* 0.001) so that, following treatment, mean LVEF in trientine-treated diabetic animals was equivalent to that in non-diabetic controls (P > 0.05).

CO increased progressively in SHAM rats during the study (*P <* 0.0001) as normal organ growth progressed, but this increase did not occur in the DIA animals (Figure 3B, Tables 1, 2 and 3). CO/LVM fell significantly in both diabetic groups between Weeks 0 and 8 (*P <* 0.0001 in each) and further still in DIA animals between Weeks 8 and 16 (*P <* 0.0001). The lack of normal increase in CO is consistent with impaired growth in diabetic animals (Figure 2B). Eight weeks trientine treatment elevated CO and CO/LVM in TETA-DIA rats to a level that was significantly higher than in SHAM rats (*P <* 0.001) although the CO of TETA-DIA animals remained significantly lower than in SHAM rats (*P <* 0.01). Trientine also improved other indexes that had previously been impaired by diabetes (Table 3), including stroke volume/LVM (*P <* 0.0001), LVESV/LVM (*P <* 0.0001) and LVEDV/LVM (*P =* 0.030). These data are consistent with drug-evoked improvements in systolic and diastolic function.

### Effects of trientine treatment on cardiac output in an ex vivo working-heart model

**Figure 4:**
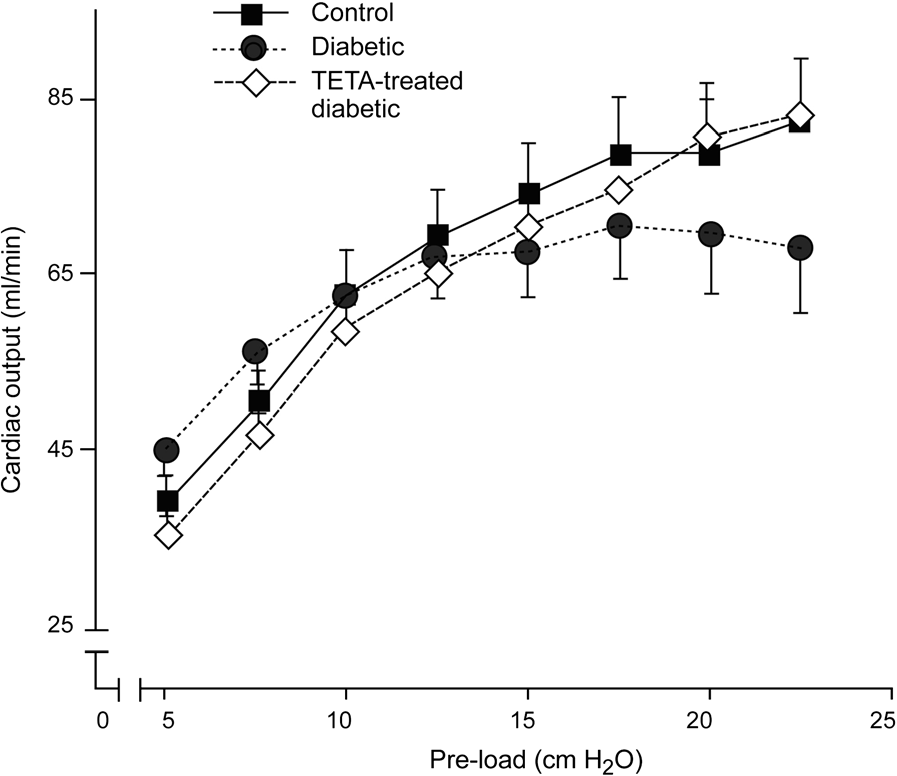
**Response of CO to increasing preload in ex vivo isolated-perfused working hearts from experimental groups of Control (n = 12), Untreated-Diabetic (n = 12) and Trientine-treated Diabetic (n = 13) adult male rats.** Abbreviation: TETA, triethylenetetramine.

We also measured ex vivo CO at increasing preload values to determine the relationship between in vivo and ex vivo estimates of this variable in the same individuals (Figure 4) [5]. Ex vivo CO-preload responses were consistent with those we have previously reported [5,7]. Increasing preload elevated CO in all groups (all *P <* 0.0001). However, CO in untreated-diabetic rats did not respond normally once preload values were increased above the peri-physiological value of ~15 cmH_2_O, as shown by flattening of the preload-CO curve above this point (CO-preload:treatment interaction term in the LME, *P <* 0.001). By contrast, the preload-CO curve in trientine-treated diabetic animals was restored to normal, and was significantly improved compared with that for untreated diabetic animals (CO-preload interaction term in LME, *P <* 0.001). These findings are consistent with the in vivo effects of trientine treatment on LVEF and CO measured in the cMRI studies presented above. Thus in vivo and ex vivo measurements of CO following trientine treatment are mutually consistent in that both have demonstrated substantive drug-mediated improvement in CO of diabetic animals.

### Time-dependent effects of trientine on cardiac function as determined by linear mixed-effects modeling

Linear mixed effects modelling (LME) “provides a generally flexible and powerful methodology for the analysis of grouped data such as repeated measures” [31], and was employed here to analyze the time-dependent effects of trientine treatment on cardiac function. Significant treatment-time interactions are considered to yield robust evidence of therapeutic efficacy in such studies [32].

Results of LME-based analysis of the effects of trientine treatment on indexes of cardiac function have been shown in Table 4. Trientine treatment exerted a modest time-dependent effect to ameliorate the effects of diabetes on body-weight (*P =* 0.035) whereas it had no measurable effect on blood glucose levels or heart rate. It significantly improved LVM/BW (*P =* 0.0077), whereas the *P-*value corresponding to the corresponding non-indexed variable, LVM, was 0.046: this contrast provides evidence that supports the use of LVM/BW as opposed to unindexed LVM to measure time-dependent therapeutic effects on LV mass. This finding is consistent with previous reports [5,12].

Trientine exerted substantive effects to improve CO, CO/LVM, LVEF, stroke volume, and stroke volume indexed to LVM (all *P <* 0.0001) (Table 4), consistent with results from the corresponding ANOVA analysis. These effects were caused by improvements in both systolic and diastolic function: effects on LVESV and LVESV/LVM (both *P <* 0.0001) were arguably more prominent than those on LVEDV (*P =* 0.0091) and LVEDV/LVM (*P =* 0.0013), which may point to a greater effect of trientine on systolic function in this study. Trientine treatment has previously been shown to exert therapeutic effects on both diastolic and systolic function in rats with diabetes-induced heart failure [5].

## Discussion

Here we have reported cMRI-derived measurements of the effects of trientine treatment on cardiac function in rats with established heart failure caused by diabetes. Effects of diabetes on cardiac function were characterized, inter alia, by substantive impairment of functional indexes including LVEF, CO/LVM (a measure of myocardial efficiency [30,33]), LVEDV/LVM, LVESV/LVM, stroke volume/LVM and elevated LVM/BW (the latter a measure of relative LV hypertrophy). These findings are consistent with prior reports and provide substantive validation of the model used in the current studies [33].

Eight weeks trientine treatment significantly improved cardiac function without modifying hyperglycemia, thus effectively breaking the link between defective glucose homeostasis and organ damage, and implicating defective copper regulation in the mechanism by which diabetes causes heart failure. Cardiac function improved in every drug-treated rat, consistent with marked trientine-evoked improvement in cardiac performance in rats with established heart failure caused by diabetic cardiomyopathy. Furthermore, the beneficial effect of trientine treatment on CO in these studies was consistent between the cross-sectional and time-dependent in vivo cMRI analyses, and the subsequent cross-sectional ex vivo endpoint measurements in the same individuals.

Thus, trientine treatment substantively improved cardiac function in this widely-used model of diabetes-evoked heart failure. Previous studies have shown that trientine treatment can improve LV hypertrophy, ex vivo indexes of diastolic and systolic function, and numerous cardiac biomarkers in animals with diabetes-induced heart failure [6,34-36]. To date, however, there has been no direct in vivo evidence that trientine can significantly improve cardiac function in animals with established heart failure. The current results are significant because they provide robust support for ongoing development of trientine treatment for the experimental treatment of diabetic patients with heart failure, and validate the use of cMRI to measure the responses of trientine treatment in diabetic patients with impaired CO and LVEF.

Different values for LVEF have been reported in the literature by different groups for normal rats or those with heart failure [33,37-39]. Between-group differences in reported values may be related to differences in rat strains (for example Sprague–Dawley vs. Wistar), methodological differences, or differences in the severity of diabetes or the time that measurements were made after the onset of disease. The values obtained in our study (Tables 1, 2 and 3) are consistent with data reported by others in the literature, as measured by cMRI [38], PET scanning [37] or echocardiography [37]. For example, LVEF values comparable to ours in normal adult rats have been reported that were determined by cMRI (79 ± 4%) [38], cardiac PET scanning (83.2 ± 8.0%) [37] and echocardiography (81.6 ± 6.0%) [37]. The latter group also reported values of LVEF in heart failure, of 54.6 ± 15.9% by PET scanning and 54.2 ± 13.3% by echocardiography that are also comparable to our measurements (Tables 2, 3, 4). By contrast, others have reported moderately lower estimates of LVEF in control (~64-67%) and diabetic (~42-45%) rats [33,39]. Thus there is considerable variability in published LVEF measurements in both normal rats and those with heart failure, and our values lie in the mid-range of reported values. In studies such as ours of the reversal of disease effects, it is probably more important that measurements in control, diabetic and drug-treated diabetic animals were made by using a consistent analytical approach, as was done here. Furthermore, our experiment employed significantly larger numbers of animals per group than were used by some of the others [33] so the power of our study was considerably greater.

Treatment with the long-acting calcium channel blocker, azelnidipine in STZ-diabetic rats has also been reported to cause significant improvements in myocyte contractile function, oxidative stress and myocardial apoptosis, which were attributed to improved myocellular calcium homeostasis [40]. However, there is compelling evidence that impaired myocellular calcium handling does not explain the impaired contractility of diabetic cardiomyopathy in STZ-diabetic rats, which is actually attributable to LV remodeling and consequential impairments in calcium responsiveness [41]. Therefore, the actual mechanism of the reported effects of azelnidipine [40] is by no means clear.

The current results extend our previous work in nonclinical models of diabetic organ damage [5,7,8,36,42,43] and in clinical trials [10,13,44,45], where we have shown that trientine is safe in both nondiabetic volunteers and diabetic patients, and that it significantly improves antioxidant defences and improves LVM in diabetic patients with LVH [6,12]. Consistent with our prior results, trientine treatment also improved diabetes-mediated cardiac damage in Zucker diabetic rats, an animal model of T2DM, whose metabolic disturbances were less severe than those in the animals we studied here [35]. Taken together with our nonclinical and clinical results, these latter independent findings are consistent with our current conclusions and provide further support for the idea that trientine treatment can improve cardiac function in diabetic individuals with different degrees of metabolic perturbation.

The current study complements and extends our previous studies in patients with T2DM and LV hypertrophy, in whom 12-months trientine treatment significantly improved LV mass indexed to body-surface area (LVM_bsa_) without adverse remodeling consequences [12]. In that study, patients had LVH and abnormal diastolic filling as demonstrated by mitral inflow Doppler with preload reduction and LV ejection fraction ≥ 45% by echocardiography, with evidence of diastolic dysfunction, and it remains to be determined whether trientine treatment can improve indexes of cardiac function in patients with established heart failure.

Previous mechanistic studies have indicated that trientine decreases LVH by a combination of beneficial effects through which it restores the integrity, inter alia, of ventricular composition, cardiac myocellular actomyosin filaments, mitochondria, and the ECM [5,7,43]. At the molecular signaling level, these responses are mediated, at least in part, by normalization of the myocardial and arterial TGF-β/SMAD signaling pathways [7] with concurrent bolstering of antioxidant defense mechanisms [36]. These beneficial effects on tissue and organ structure/function are probably mediated through trientine-evoked normalization of copper regulation [6-8] with resulting lowering of oxidative stress, and accompanying improvements in ROS metabolism and mitochondrial function [36,43].

Of potential relevance to the current findings are results from treatment with another copper chelator, tetrathiomolybdate (TTM) that can inhibit acute inflammatory responses in vivo [46]. In a recent study, TTM reportedly inhibited vascular inflammation and atherosclerotic lesion development in apolipoprotein-E deficient mice [47], raising the possibility that similar effects could also contribute to the cardiovascular responses to trientine. However, in this study, 10-weeks treatment with TTM induced significant lowering of circulating ceruloplasmin levels, raising the possibility that the reported findings could have been confounded by concomitant copper deficiency, as signaled by marked lowering of serum ceruloplasmin levels. By contrast, under the conditions used in this study, trientine does not cause copper deficiency [5,12].

What information is currently available about the dosage and tolerability of trientine in diabetes? In previous nonclinical studies [5,7,34,35], trientine dosages have been equivalent on a mg-per-kg basis in rats to those employed in the current study and in our recently reported 12-month clinical trial [12]. Trientine has been well tolerated by diabetic and non-diabetic rats alike in previous studies, in which it has typically been administered orally in the drinking water for periods of between 6 and 8 weeks. It has also been well-tolerated by diabetic patients in recent Phase 2 studies in one of which it was administered at 1200 mg/d for 12 months [6,12]. Furthermore, the trientine-treated patients in these studies did not develop copper deficiency, as shown by levels of informative biomarkers [10,12], namely serum copper and ceruloplasmin [48]. Equivalent and higher doses were also well tolerated by the non-diabetic volunteers in our two recent Phase 1 pharmacokinetic-pharmacodynamic studies [10,13]. In addition, comparable trientine dosages have been used successfully for many years in the chronic treatment of patients with Wilson’s disease [17,18]. An optimal trientine dosage for the treatment of heart failure in diabetic patients is likely to lie in the region of 1200–2400 mg/day (600–1200 mg b.i.d.) [6,10,12,13].

The current study has limitations. Trientine treatment significantly improved cardiac function in diabetic rats with established heart failure. Nevertheless, although substantively improved, their cardiac function was still markedly impaired after eight weeks drug treatment compared with non-diabetic controls. In comparison, T2DM patients with LVH displayed on average a ~50% lowering of elevated LVM_bsa_ values after 12-months trientine treatment [12]. Moreover, although CO was apparently fully restored in this ex vivo study, comparison with the cMRI-based measurements shows that the level of in vivo recovery was less, at about ~30%. This difference may well reflect greater sensitivity of the in vivo cMRI-based measurements. Further studies will be required to determine the maximal restoration of CO, CO/LVM, LVEF and stroke volume/LVM values achievable by longer periods of trientine treatment, and what length of treatment might be required to achieve an optimal response. In addition, the long-term sustainability of trientine-evoked improvements in cardiac function also remains to be established. Finally, it remains to be determined whether improvements in functional parameters might herald lowered rates of clinically-relevant cardiac endpoints and increased survival in patients with diabetes-associated heart failure.

Our measurements of myocardial fat indicate that the model of severe STZ-evoked diabetes we employed here is unlikely to yield useful information about copper/trientine-myocardial lipid interactions. We have employed the STZ-mediated model of diabetes-induced heart failure because it is reproducible and consistent in terms of time of onset, progression, and severity, and its cardiac structure/function responses are clearly consistent with those in diabetic patients [5,12]. However, the metabolic disturbance in this model is more akin to that in patients with severe T1DM rather than T2DM, where the metabolic disturbance is usually less severe. Trientine treatment did not modify plasma lipid levels in rats with hypercholesterolemia and hypertriglyceridemia caused by diabetes [36]. We conclude that effects of trientine on myocardial metabolism will need to be studied in other nonclinical models of hyperlipidemic diabetes, such as the Zucker diabetic rat, in which myocardial lipid levels are elevated in untreated diabetic animals, in order to better understand its effects on lipid regulation.

Our measurements of simultaneous copper and triethylenetetramine concentrations in diabetic animals and humans undergoing trientine treatment confirm that the molar trientine free-base to Cu(II) ratio is consistently > > 1 in the urine [14,44]. These findings, taken together with the strong, selective binding of triethylenetetramine to Cu(II) [9,11], are consistent with protection by the drug against any copper toxicity that could otherwise be caused in the body during trientine-mediated extraction of copper into the urine. Trientine can thus effectively suppress Cu(II)-mediated cytotoxicity. Biological effects that may explain trientine’s therapeutic efficacy include its ability to elicit structural and functional improvements in the cardiac myocytes [5], the extracellular matrix [7] and cardiac mitochondria [43], and to suppress oxidative stress, including that in cardiovascular and renal tissues [8,13,36,49]. Current data strongly support the view that trientine’s efficacy is underpinned in substantive part by its ability to suppress Cu(II)-mediated oxidative stress and mitochondrial dysfunction [36,43].

Administration of trientine and measurement of the resulting urinary copper excretion unmasks the extent of the copper overload in diabetes [5,6,12,14,45]. For example, an oral challenge with trientine in diabetic patients revealed a significant increase in chelated Cu(II) [5]. The presence of elevated systemic copper following trientine administration is an important sign because such copper is catalytically active and therefore (presumably) capable of catalyzing the formation of highly-destructive hydroxyl radicals from substrates such as superoxide anion or hydrogen peroxide [9,22]. Probing of diabetic animals and patients with various applicable types of spectroscopy, including atomic absorption, atomic emission, ICP*-*MS, EPR and particle-induced X-ray emission (PIXE), has demonstrated the presence of copper excess in the whole body [5,6,12], urine, coronary arteries [5], and kidneys [8]. Thus, defective copper regulation could well make a major contribution to the causation of oxidative stress in diabetes, which can thus be suppressed by treatment using trientine with the outcome that organ damage is thus substantively ameliorated.

How might particular organs be selectively targeted by copper-mediated oxidative stress? Elevated pathogenetic tissue binding of copper occurs in diabetes [5,6]. This phenomenon could well be caused by copper-catalyzed ‘glycoxidation’ that mediates formation of advanced glycation end-products (AGEs) and consequential AGE-modification of susceptible amino-acid residues, particularly lysine, arginine, histidine and cysteine, in long-lived fibrous proteins such as connective-tissue collagens [5,50-54]. Such AGE-modified amino-acid residues are thought to act as endogenous chelators [9,21,55] that can increase the copper content of organs such as the coronary arteries and kidneys by binding increased amounts of catalytically-active Cu(II), thereby focusing the related oxidative stress into susceptible tissues. The pathogenetic significance of these phenomena was not apparent before we demonstrated suppression of diabetes-mediated organ damage by Cu(II)-selective chelation [5-8,12,42,43].

What biological properties of copper underpin its pathogenetic behaviour in diabetes? Most intracellular copper is tightly bound and regulated by copper-binding proteins [56], and intracellular free copper is essentially undetectable [57]. Cu(II), which is present in urine from drug-treated diabetic rats, is the most effective divalent ion for binding to organic molecules and the main extracellular copper ion, whereas Cu(I) predominates inside cells [22]. Trientine binds Cu(II) less strongly than most physiological copper-binding proteins [22]. These observations, taken together with our demonstration of the prompt increase in Cu(II) excretion after oral trientine administration in diabetic patients [5,6], and after injection into the coronary arteries in ex vivo cardiac preparations from diabetic rats [5], indicate that this increased Cu(II) is unlikely to be released acutely from an intracellular pool. More likely, the Cu(II) is bound to extracellular matrix (ECM) components, such as collagen; because it is readily extracted by trientine, the increased Cu(II) must be loosely bound and is therefore the probable cause of the observed increase in oxidative stress suppressible by trientine [36]. Importantly, consistent with our recent X-ray crystallographic studies, triethylenetetramine-binding to Cu(II) can suppresses its catalytic activity, thus protecting the renal tubules from the toxicity that could otherwise ensue.

There are several strong endogenous copper chelators including spermine, spermidine and carnosine, which are localized mainly within cells. There are no available data known to us concerning their ability to extract systemic copper into the urine in diabetes, or to treat diabetic cardiomyopathy. Spermine was reportedly highly toxic when administered to chicks [58] and long-term studies of its tolerability on administration to diabetic rats are currently unavailable. Based on their structures and related thermodynamic considerations, putrescine (a diamine) and spermidine (a triamine) cannot bind copper as strongly as triethylenetetramine (a tetraamine). Much research would be required before the putative utility of any of these endogenous polyamines in the treatment of diabetic heart disease could be ascertained.

Some studies with clinically significant drugs used to treat diabetic patients, such as metformin, pioglitazone and some of the ARBs, have suggested that they are chelators that could exert their therapeutic effects through copper chelation [59,60]. The strength of their copper-binding ability appears to be relatively weak compared with that of triethylenetetramine based on currently available evidence. Moreover, there is no available in vivo evidence to indicate that any of them acts by correcting the dysregulated copper homeostasis that occurs in diabetes [6].

Mechanisms of action of trientine and metformin are now briefly compared with respect to roles of copper. Metformin is an anti-hyperglycemic biguanide drug that is used extensively in the treatment of type 2 diabetes [61]. It acts via AMP-activated protein kinase (AMPK), whose activation is required for metformin’s inhibitory effect on hepatic glucose production via inhibition of the diabetes-mediated activation of the gluconeogenic genes phosphoenolpyruvate carboxykinase and glucose-6-phosphatase [62-64]. The idea has recently been advanced that metformin might function through binding to copper in locations such as mitochondria [59]. As has been discussed above, there is a substantive body of evidence linking defective copper homeostasis to the pathobiology of diabetes and its experimental treatment with trientine [9,21] which points to major differences between the mechanisms of action of the two drugs. For example, trientine does not lower blood glucose in diabetic rats or patients, whereas glucose lowering is metformin’s major therapeutic effect [5,12]. Moreover, in a study comparing patients with type-2 diabetes and LV hypertrophy, who were taking standard therapeutic doses of metformin with or without trientine, LV hypertrophy was unchanged in those taking metformin only, but was substantively improved in the trientine-treated patients [12]. Furthermore, trientine acts in vivo as a chelator that removes excess Cu(II) from the body, whereas patients treated with metformin have rates of urinary copper excretion similar to basal (that is, untreated) values in diabetic patients [6,12]. Therefore, any role played by copper binding in metformin’s mode of action must be very different from that it plays for trientine.

In conclusion, eight weeks trientine treatment caused significant improvement in numerous indexes of cardiac function as determined by cMRI in a widely employed nonclinical model of diabetes-induced heart failure, the STZ-diabetic rat. These findings support and extend previous studies in diabetic rats, and are consistent with results from a recent Phase 2 trial of trientine treatment in diabetic patients with LVH. The Cu(II)-selective chelator trientine is the first in a new class of orally-active molecules with application in the experimental pharmacotherapy of the diabetic complications. The evidence presented here supports the further clinical investigation of trientine treatment in patients with diabetes and heart failure.

### Endnote

^a^Abbreviations: AGEs, advanced glycation endproducts; ANOVA, analysis of variance; ARB, angiotensin II receptor blocker; BW, body-weight; cMRI, cardiac magnetic-resonance imaging; CI, 95% confidence interval of the mean; CO, cardiac output; Cu(I), univalent copper; Cu(II), divalent copper; ECM, extracellular matrix; EPR, electron paramagnetic resonance spectroscopy; LME, linear mixed-effects; LVEDV, LV end-diastolic volume; LVEF, left-ventricular ejection fraction; LVESV, LV end-systolic volume; LVM, LV mass; PET, positron-emission tomography; PIXE, particle-induced X-ray emission spectroscopy; RBS, Rutherford backscattering spectroscopy; STZ, streptozotocin; T1DM, type-1 diabetes; T2DM, type-2 diabetes; TE, echo time; TR, repetition time; TTM, tetrathiomolybdate.

## Competing interest

G.J.S. Cooper is an honorary consultant to PhilERA, holder of patent rights to trientine for the treatment of diabetes and related metabolic diseases. All other authors declare no duality of interest.

## Authors’ contributions

JL designed, performed and interpreted research, and drafted the manuscript; BP designed and interpreted research and revised the manuscript; SP supervised and interpreted research and reviewed the manuscript; SC performed and interpreted research and revised the manuscript; ML performed research and reviewed the manuscript; HX designed research and reviewed the manuscript; GG performed and interpreted research and reviewed the manuscript; AP designed and supervised research and revised the manuscript; BC designed and interpreted research and revised the manuscript; AY designed, supervised and interpreted research and revised the manuscript; GC designed, supervised, and interpreted research, and drafted the manuscript. All authors have seen and approved the manuscript.
